# Supplementary material for: Functional but not obligatory link between microsaccades and neural modulation by covert spatial attention
Source: Nat Commun. 2022 Jun 17;13:3503. doi: 10.1038/s41467-022-31217-3 (PMC9205986; doi:10.1038/s41467-022-31217-3)
Supplement: Supplementary file 1 — Supplementary Information [file 41467_2022_31217_MOESM1_ESM.pdf]

SUPPLEMENTARY INFORMATION

(Supplementary Figures S1-S7 and supplementary results text)

Supplementary information associated with the manuscript:

title: Functional but not obligatory link between microsaccades and neural modulation by covert spatial attention
authors: Baiwei Liu, Anna C Nobre, Freek van Ede
journal: Nature Communications
year: 2022
contact: Baiwei Liu (b.liu@vu.nl); Freek van Ede (freek.van.ede@vu.nl)

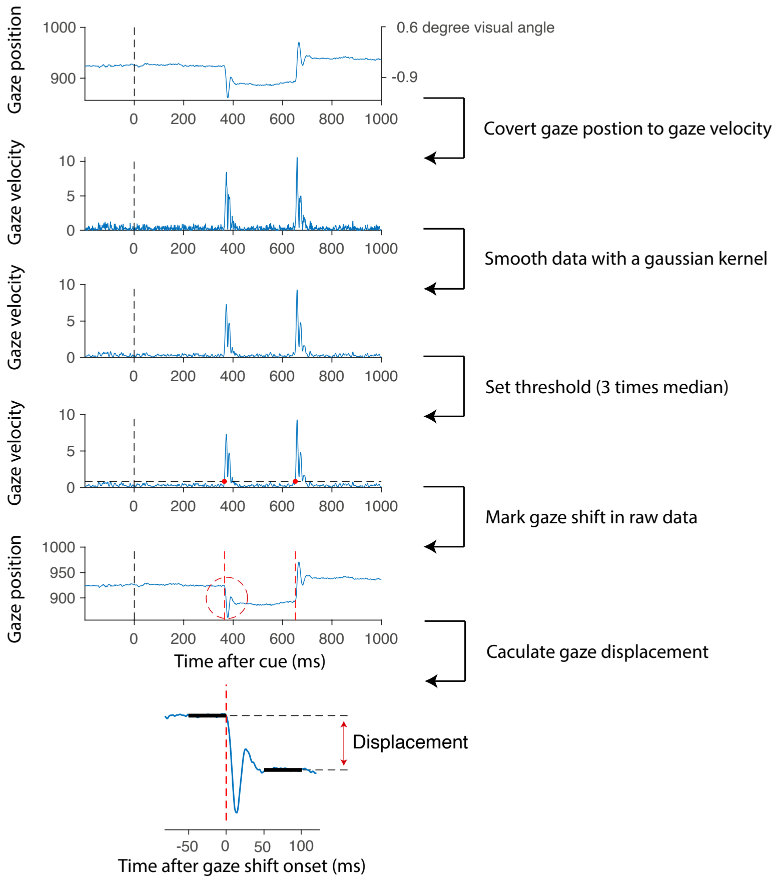

Supplementary Figure 1. Analysis pipeline for detecting horizontal gaze shifts. We set a relatively low threshold to ensure we would not wrongfully misclassify ‘no-microsaccade’ trials as the results of a too high threshold. After shift detection we discarded all shifts with a displacement smaller than 1% (0.057 degrees visual angle). We further discarded all trials with a blink occurring in the 200-600 ms selection window of interest. To separate our three trial classes of interest (Fig. 1d) we thus only considering trials with usable eye trace in the window of interest in which either no gaze shift was detected, or in which a gaze shift was detected with a displacement of at least 0.057 degrees visual angle. We focused exclusively on horizontal gaze shifts, provided our goal to link directional left/right biases in gaze with modulation of spatial attention associated with the left/right attended location.

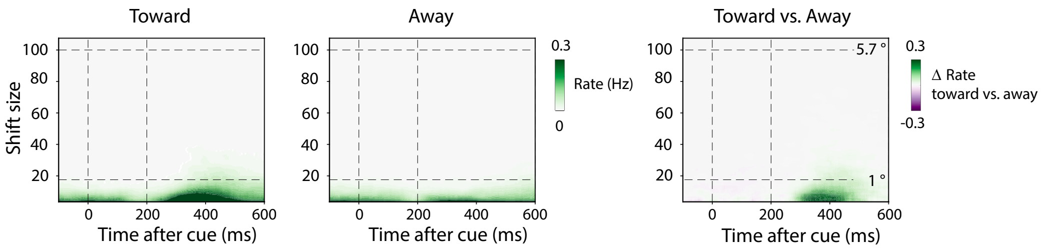

Supplementary Figure 2. Gaze-shift rates as a function of gaze-shift magnitudes, split by microsaccade direction. Data on the right as in main Figure 1c. The left two panels show these data separately for toward and away gaze shifts (relative to the memorised location of the cued memory item).

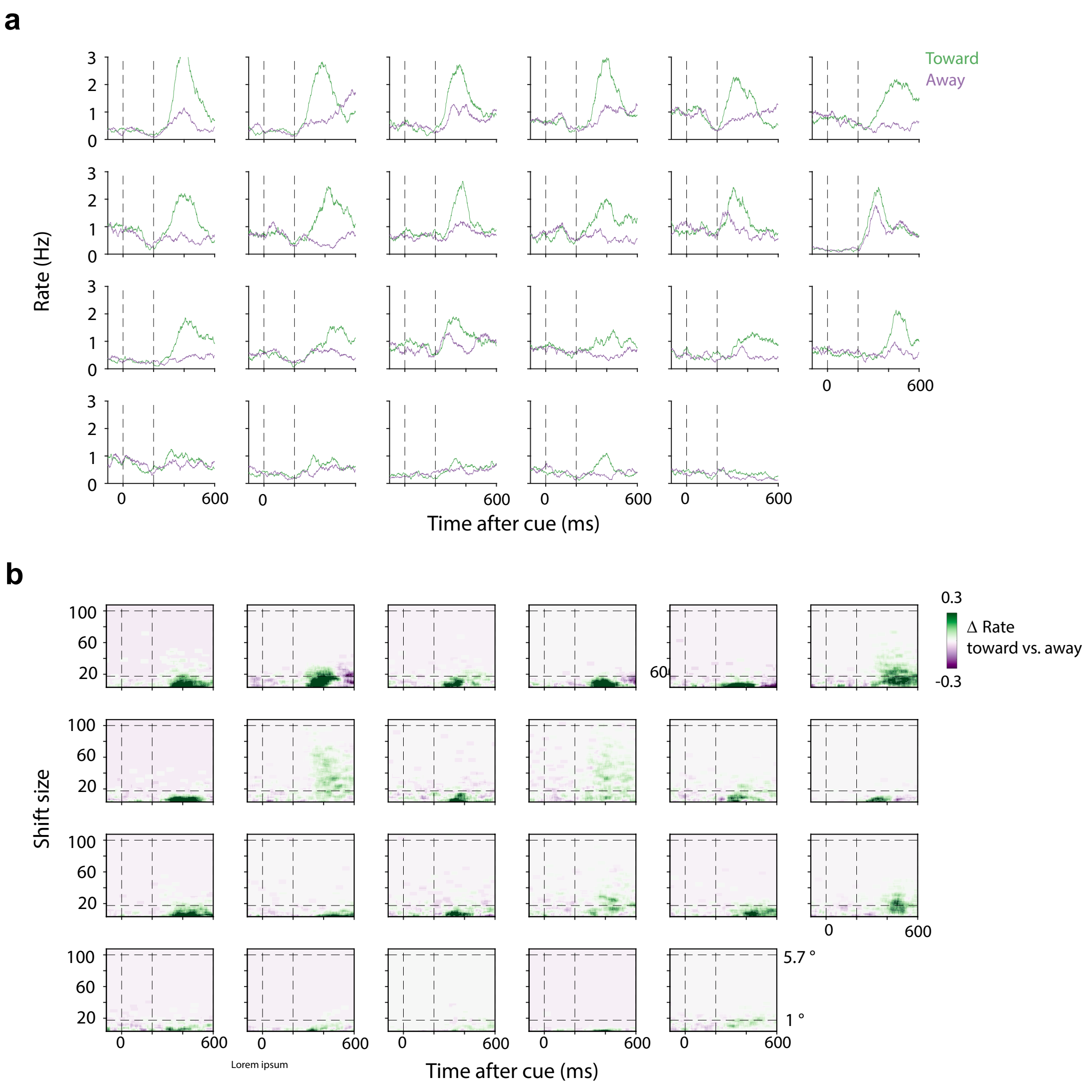

Supplementary Figure 3. Directional biases in microsaccades are visible in every participant. Data as in group-level data in main Figure 1, panels b and c. Each panel represents an individual participant. For visualization purposes, we sorted participants by their proportion of toward-microsaccade trials.

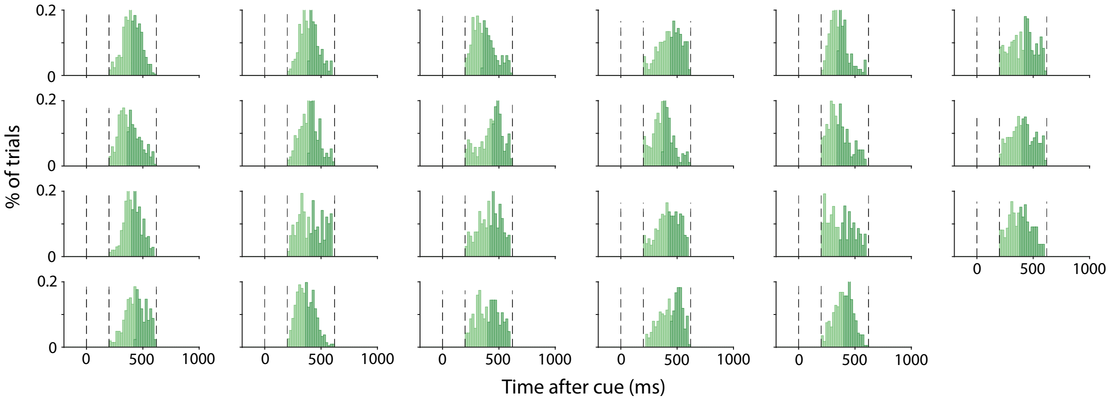

Supplementary Figure 4. Participant-specific distributions of microsaccade onset latencies in trials with early and late microsaccades (median split per participant). Accumulated group-level distributions in main Figure 4A.

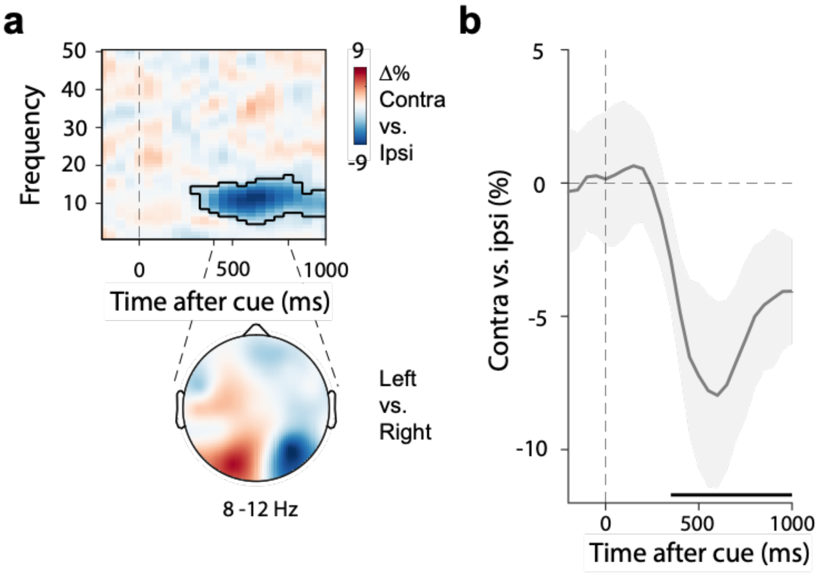

Supplementary Figure 5. Trials with no microsaccade in the 0-600 ms post-cue window also show preserved alpha modulation by covert spatial attention. a) Neural lateralisation in visual electrodes relative to the memorised location of the memory item after the selection cue (top) together with the associated topographical map of the difference in 8-12 Hz alpha power in the 400-800 ms interval between trials in which to-be-reported memory item was left vs. right at encoding (bottom). Black outline indicates significant cluster (two-sided cluster-based permutation test: $p < 0.001$). **b)** Averaged 8-12 Hz alpha lateralization. Black horizontal line indicates significant temporal cluster (cluster $p < 0.001$). Time courses show mean values, with shading indicating 95% CI (calculated across 23 participants).

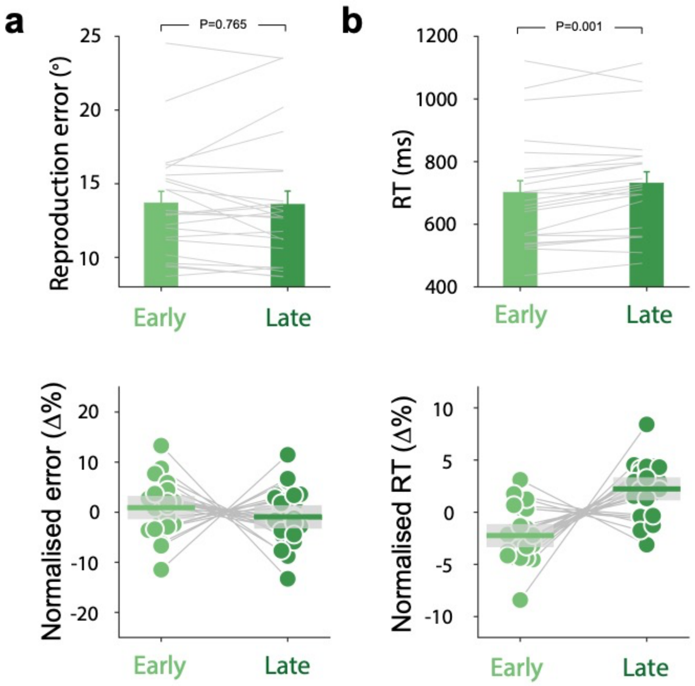

Supplementary Figure 6. Trials in which the first toward microsaccade after the cue occurs early (vs late) have faster onsets (but not lower error) of the memory-guided report. Top panels show mean of raw performance with grey lines indicating individual-participant data, while the bottom panels show mean of normalised performance (percent change from mean) together with individual datapoints. Error bars in the top panels indicate ±1 SEM. Shadings in the bottom panels indicate 95% confidence interval. Both the SEM and confidence interval are calculated across participants (n = 23). Responses were initiated faster after the onset of the memory cue in trials in which the first-detected toward microsaccade was early vs late: $t(22) = 3.69$, $P = 0.001$, $d = 0.769$. The statistical tests used here were two-sided paired samples t-test.

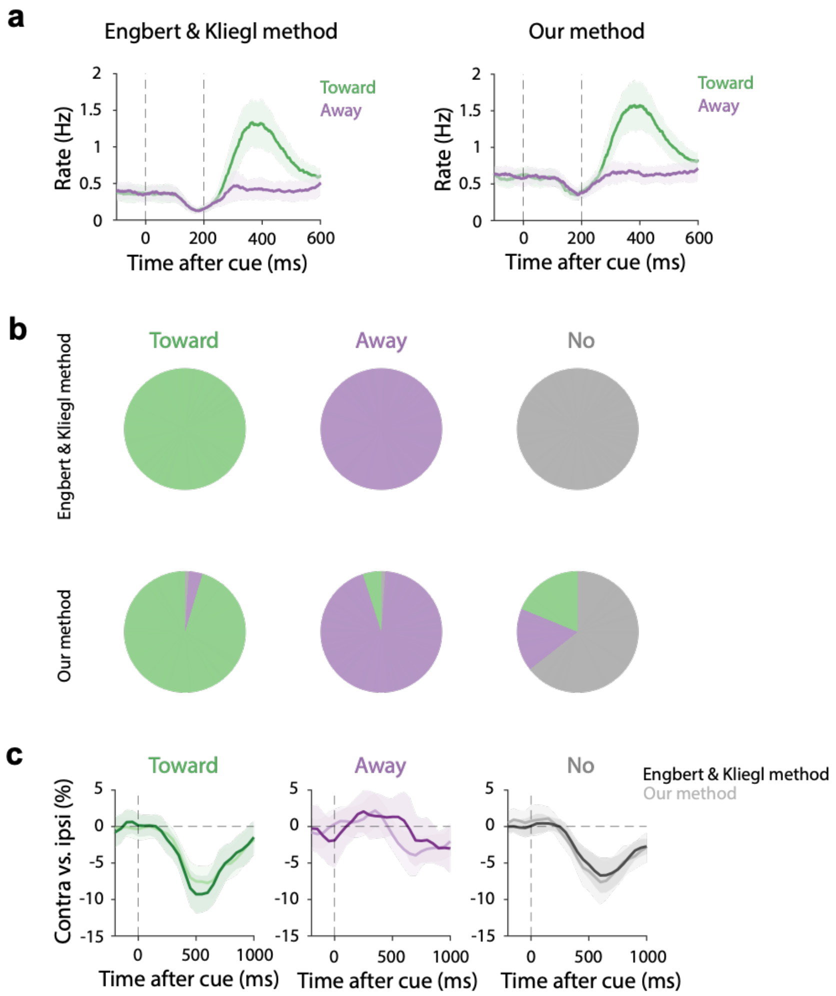

Supplementary Figure 7. Replication of our main results using an alternative microsaccade-detection method. a) Time courses of gaze-shift rates (number of microsaccades per second) for shifts toward and away from the memorised location of the cued memory item. The left panel shows the results from Engbert & Kliegl's [20] method; the right panel shows the results from our method. **b)** The overlap of trials in each condition that sorted by Engbert & Kliegl's method and our method. The top row represent the trials sorted by Engbert & Kliegl's method while the bottom row represent the overlap in trial-allocation when relying on our method. **c)** In each condition, the overlays of averaged 8-12 Hz alpha lateralization from the trials that are sorted by Engbert & Kliegl's method and by our method. Time courses show mean values, with shading indicating 95% CI (calculated across 23 participants).

Supplementary results text associated with Supplementary Figure 7

While we titrated our method of microsaccade detection to our current aims (deliberately using a relatively low threshold for detecting, and focusing on horizontal shifts of gaze), we obtained similar results when using another, more established, method by Engbert & Kliegl [20] instead.

First, we obtained similar temporal profiles of microsaccades and their modulation by internal selective attention (Supplementary Fig. 7a).

Second, we converged on strongly overlapping trial classifications when relying on our method compared to the Engbert & Kliegl method. When the Engbert & Kliegl method detected a toward microsaccade in the 200-600 ms post-cue window of interest, so did our method in the vast majority of trials (Supplementary Fig. 7b, left). Likewise, when the Engbert & Kliegl method detected an away microsaccade, so did our method (Supplementary Fig. 7b, middle). Moreover, when the Engbert & Kliegl method did not detect a microsaccade (in our window of interest), our method occasionally still detected a toward or away microsaccade (Supplementary Fig. 7b, right). This reveals that our method was more strict when it comes to allocating the label “no-microsaccade” to a trial (i.e. our methods seemed more liberal for allocating gaze shifts, and thereby more conservative for determining no-microsaccade trials). This is an important characteristic given our central aim of addressing whether the neural modulation is preserved in these critical no-microsaccade trials.

Finally, irrespective of these slight differences in trial-allocation between methods, alpha modulation in each of the three trial classes was highly comparable between the two microsaccade-detection methods (Supplementary Fig. 7c) – revealing a clear alpha modulation in the no-microsaccade trials in both cases. These data thus help validate our method, and reinforce our central conclusions by showing that our main findings generalise when using a different microsaccade-detection method.
