# Supplementary material for: Functional but not obligatory link between microsaccades and neural modulation by covert spatial attention
Source: Nat Commun. 2022 Jun 17;13:3503. doi: 10.1038/s41467-022-31217-3 (PMC9205986; doi:10.1038/s41467-022-31217-3)
Supplement: Supplementary file 3 — Reporting Summary [file 41467_2022_31217_MOESM3_ESM.pdf]

nature portfolio

Corresponding author(s): Baiwei Liu, Dr Freek van Ede

Last updated by author(s): May 3, 2022

Reporting Summary

Nature Portfolio wishes to improve the reproducibility of the work that we publish. This form provides structure for consistency and transparency in reporting. For further information on Nature Portfolio policies, see our Editorial Policies and the Editorial Policy Checklist.

Statistics

For all statistical analyses, confirm that the following items are present in the figure legend, table legend, main text, or Methods section.

n/a	Confirmed	
☐	☒	The exact sample size (*n*) for each experimental group/condition, given as a discrete number and unit of measurement
☒	☐	A statement on whether measurements were taken from distinct samples or whether the same sample was measured repeatedly
☐	☒	The statistical test(s) used AND whether they are one- or two-sided<br>*Only common tests should be described solely by name; describe more complex techniques in the Methods section.*
☒	☐	A description of all covariates tested
☐	☒	A description of any assumptions or corrections, such as tests of normality and adjustment for multiple comparisons
☐	☒	A full description of the statistical parameters including central tendency (e.g. means) or other basic estimates (e.g. regression coefficient) AND variation (e.g. standard deviation) or associated estimates of uncertainty (e.g. confidence intervals)
☐	☒	For null hypothesis testing, the test statistic (e.g. F, t, r) with confidence intervals, effect sizes, degrees of freedom and P value noted<br>*Give P values as exact values whenever suitable.*
☒	☐	For Bayesian analysis, information on the choice of priors and Markov chain Monte Carlo settings
☒	☐	For hierarchical and complex designs, identification of the appropriate level for tests and full reporting of outcomes
☐	☒	Estimates of effect sizes (e.g. Cohen's *d*, Pearson's *r*), indicating how they were calculated

Our web collection on statistics for biologists contains articles on many of the points above.

Software and code

Policy information about availability of computer code

Data collection	Neurobs Presentation version 18.3 07.18.16<br>EyeLink 1000<br>Neuroscan SCAN version 4.5
Data analysis	FieldTrip version 20190129; Matlab version 2021a; Codes (https://github.com/BaiweiLiu/VWMMS_EEG_alpha-covertAttention/releases/tag/v.1)

For manuscripts utilizing custom algorithms or software that are central to the research but not yet described in published literature, software must be made available to editors and reviewers. We strongly encourage code deposition in a community repository (e.g. GitHub). See the Nature Portfolio guidelines for submitting code & software for further information.

Data

Policy information about availability of data

All manuscripts must include a data availability statement. This statement should provide the following information, where applicable:

- Accession codes, unique identifiers, or web links for publicly available datasets
- A description of any restrictions on data availability
- For clinical datasets or third party data, please ensure that the statement adheres to our policy

All data are publicly available through the Dryad Digital Repository. The behavioural and eye-tracking data can be freely downloaded at: https://doi.org/10.5061/dryad.m99r286 (Experiment 1). The corresponding EEG data can be freely downloaded at: https:// doi.org/10.5061/dryad.sk8rb66.

Field-specific reporting

Please select the one below that is the best fit for your research. If you are not sure, read the appropriate sections before making your selection.

☒ Life sciences ☐ Behavioural & social sciences ☐ Ecological, evolutionary & environmental sciences

For a reference copy of the document with all sections, see nature.com/documents/nr-reporting-summary-flat.pdf

Life sciences study design

All studies must disclose on these points even when the disclosure is negative.

Sample size	Twenty-five healthy human volunteers participated in the experiment (age 19-36; 11 male, 2 left handed). The sample size was set based on the planned EEG study, in which the sample size was chosen based on previous publications from our lab (e.g. ref. 40) that had similar designs and focused on similar neural signatures.
Data exclusions	Two participants were excluded due to the poor quality of their eye-tracking data (as also reported in our previous dedicated eye-tracking article).
Replication	Our main results replicated when using a complementary microsaccade-detection method (ref. 20).
Randomization	The study did not contain experimental groups but followed as within-subjects design. Probed item location was always randomised across trials. Before randomisation, all conditions of interest were set to have equal trial numbers.
Blinding	Data collection and analysis were not performed blind to the conditions of the experiments. However, trial removal and microsaccade detection were performed without knowledge of the condition to which individual trials belonged. Because there were no experimental groups, blinding at the level of group membership was not relevant.

Reporting for specific materials, systems and methods

We require information from authors about some types of materials, experimental systems and methods used in many studies. Here, indicate whether each material, system or method listed is relevant to your study. If you are not sure if a list item applies to your research, read the appropriate section before selecting a response.

Materials & experimental systems

n/a	Involved in the study	
☒	☐	Antibodies
☒	☐	Eukaryotic cell lines
☒	☐	Palaeontology and archaeology
☒	☐	Animals and other organisms
☐	☒	Human research participants
☒	☐	Clinical data
☒	☐	Dual use research of concern

Methods

n/a	Involved in the study	
☒	☐	ChIP-seq
☒	☐	Flow cytometry
☒	☐	MRI-based neuroimaging

Human research participants

Policy information about studies involving human research participants

Population characteristics	Twenty-five healthy human volunteers participated in the experiment (age 19-36; 11 male, 2 left handed). All participants had normal or corrected-to-normal vision. Nearly all participants were undergraduate students at the University of Oxford or at Brookes University. Because this was a within-subjects design, none of these variables constituted a relevant co-variate.
Recruitment	Participants were recruited through flyers and an online participant database (SONA) at the University of Oxford. There was no selection bias by the experimenters. The only potential 'bias' is that the vast majority of participants were university students (as is commonly the case in cognitive neuroscience studies).
Ethics oversight	the Central University Research Ethics Committee of the University of Oxford

Note that full information on the approval of the study protocol must also be provided in the manuscript.
